# TrkB-mediated neuroprotection in female hippocampal neurons is autonomous, estrogen receptor alpha-dependent, and eliminated by testosterone: a proposed model for sex differences in neonatal hippocampal neuronal injury

**DOI:** 10.1186/s13293-024-00596-1

**Published:** 2024-04-02

**Authors:** Vishal Chanana, Dila Zafer, Douglas B Kintner, Jayadevi H Chandrashekhar, Jens Eickhoff, Peter A Ferrazzano, Jon E Levine, Pelin Cengiz

**Affiliations:** 1 Waisman Center, University of Wisconsin, Madison, WI USA; 2 Department of Pediatrics, Division of Pediatric Critical Care Medicine, University of Wisconsin, 1500 Highland Ave - T505, Madison, WI 53705-9345 USA; 3 University of Illinois at Urbana-Champaign, Champaign, IL USA; 4 Department of Statistics and Bioinformatics, University of Wisconsin School of Medicine and Public Health, Madison, WI USA; 5 Department of Neuroscience, University of Wisconsin, Madison, WI USA; 6 Wisconsin National Primate Research Center, Madison, WI USA

**Keywords:** Neurotrophin receptor, Estrogen receptor alpha, Neonatal, Hypoxia ischemia, 7,8-dihydroxyflavone, Tyrosine kinase B receptor

## Abstract

**Background:**

Neonatal hypoxia ischemia (HI) related brain injury is one of the major causes of learning disabilities and memory deficits in children. In both human and animal studies, female neonate brains are less susceptible to HI than male brains. Phosphorylation of the nerve growth factor receptor TrkB has been shown to provide sex-specific neuroprotection following in vivo HI in female mice in an estrogen receptor alpha (ERα)-dependent manner. However, the molecular and cellular mechanisms conferring sex-specific neonatal neuroprotection remain incompletely understood. Here, we test whether female neonatal hippocampal neurons express autonomous neuroprotective properties and assess the ability of testosterone (T) to alter this phenotype.

**Methods:**

We cultured sexed hippocampal neurons from ERα^+/+^ and ERα^−/−^ mice and subjected them to 4 h oxygen glucose deprivation and 24 h reoxygenation (4-OGD/24-REOX). Sexed hippocampal neurons were treated either with vehicle control (VC) or the TrkB agonist 7,8-dihydroxyflavone (7,8-DHF) following in vitro ischemia. End points at 24 h REOX were TrkB phosphorylation (p-TrkB) and neuronal survival assessed by immunohistochemistry. In addition, i*n vitro* ischemia-mediated ERα gene expression in hippocampal neurons were investigated following testosterone (T) pre-treatment and TrkB antagonist therapy via q-RTPCR. Multifactorial analysis of variance was conducted to test for significant differences between experimental conditions.

**Results:**

Under normoxic conditions, administration of 3 µM 7,8-DHF resulted an ERα-dependent increase in p-TrkB immunoexpression that was higher in female, as compared to male neurons. Following 4-OGD/24-REOX, p-TrkB expression increased 20% in both male and female ERα^+/+^ neurons. However, with 3 µM 7,8-DHF treatment p-TrkB expression increased further in female neurons by 2.81 ± 0.79-fold and was ERα dependent. 4-OGD/24-REOX resulted in a 56% increase in cell death, but only female cells were rescued with 3 µM 7,8-DHF, again in an ERα dependent manner. Following 4-OGD/3-REOX, ERα mRNA increased ~ 3 fold in female neurons. This increase was blocked with either the TrkB antagonist ANA-12 or pre-treatment with T. Pre-treatment with T also blocked the 7,8-DHF- dependent sex-specific neuronal survival in female neurons following 4-OGD/24-REOX.

**Conclusions:**

OGD/REOX results in sex-dependent TrkB phosphorylation in female neurons that increases further with 7,8-DHF treatment. TrkB phosphorylation by 7,8-DHF increased ERα mRNA expression and promoted cell survival preferentially in female hippocampal neurons. The sex-dependent neuroprotective actions of 7,8-DHF were blocked by either ANA-12 or by T pre-treatment. These results are consistent with a model for a female-specific neuroprotective pathway in hippocampal neurons in response to hypoxia. The pathway is activated by 7,8-DHF, mediated by TrkB phosphorylation, dependent on ERα and blocked by pre-exposure to T.

## Highlights

Following in vitro ischemia, the nerve growth factor receptor TrkB is activated in the presence of the TrkB agonist 7,8-DHF only in female and not in male cultured hippocampal neurons, leading to increased neuronal survival.

Expression of ERα is increased following in vitro ischemia in female but not male hippocampal neurons.

The female hippocampal neuronal specific responses to in vitro ischemia are blocked by pre-treatment with testosterone.

The data support a model for a female-specific a neuroprotective pathway in hippocampal neurons. The pathway is activated by a TrkB agonist, dependent on ERα and blocked by testosterone.

## Abstract (significants)

In the clinical setting, it is well known that in neonates who suffer brain injury due to lack of blood flow and oxygen to the brain, the resulting damage is less severe in females compared to males. Furthermore, males sustaining brain injuries are more likely to exhibit learning and memory deficits as they mature. However, the underlying cellular mechanisms that lead to these sex differences in brain injury outcomes are poorly understood. In this report, we cultured hippocampal neurons from neonatal female and male mice and subjected them to reduced oxygen and glucose to mimic neonatal hypoxia ischemia. We found that the nerve growth factor receptor TrkB in females, as compared to males, is activated following the insult and more responsive to an exogenous nerve growth factor which leads to cell survival. In addition, expression of the estrogen receptor alpha is increased following the insult in females but not in males. Interestingly, the nerve growth factor receptor response in females is dependent on the presence of the estrogen receptor alpha. Both the nerve growth factor response and the increase in estrogen receptor alpha are abolished in females when treated with the male androgen, testosterone. Thus, our results support a model for a female-specific neuroprotective pathway in hippocampal neurons. The pathway is activated by nerve growth factors, dependent on estrogen receptor alpha, and is likely rendered inoperative in males by exposure to neonatal testosterone.

## Background

Hypoxic ischemic encephalopathy in human neonates is an important cause of life-long mortality and morbidity [1, 2]. Clinical and experimental studies suggest that female newborn brains are relatively resistant to the detrimental effects of hypoxia and ischemia (HI) while male newborn brains are more susceptible [3, 4]. In addition, male infants are at increased risk for neurodevelopmental deficits following neonatal HI including autism, learning and memory deficits and speech delays [3–6]. However, the underlying cellular mechanisms that lead to sexually differentiated outcomes following neonatal HI are largely unknown.

Neurotrophins are growth factors that regulate development and maintenance of the central nervous system, and much attention has been focused on the putative roles of neurotrophins and their receptors in neuroprotection and recovery from injury following adult stroke and neonatal HI [7–9]. Neurotrophins, such as brain derived neurotropic factor (BDNF), have been shown to phosphorylate tyrosine kinase B receptors (TrkB) in the hippocampi, reduce the infarct volume by 55% [10] and improve spatial learning following neonatal HI [11]. However, clinical trials of neurotrophin therapy for various neurological diseases have been universally disappointing [12]. Consequently, non-peptide, small molecule ligands capable of activating TrkB signaling with a high specificity and potency, and improved bioavailability have been developed [13]. Recently, a selective TrkB agonist, 7,8-dihydroxyflavone (7,8-DHF), was identified to cross the blood brain barrier with high bioavailability [9, 14, 15]. We have found that 7,8-DHF exerts a profound early and late neuroprotective effect in neonatal mice following in vivo HI but only in female mice [7, 8]. Thus, 7,8-DHF treatment reveals a sex difference in neurotrophin receptor-mediated neuroprotection following in vivo neonatal HI.

Studies suggest significant interaction between the estrogen receptor alpha (ERα) and neurotrophin receptor signaling. ERα and BDNF highly co-localized in pyramidal cells of CA1 and CA3 hippocampal subregions of intact male and female rat on postnatal day10 [16]. ERα cross talk with growth factors has been proven to be essential for neurite growth and synapse formation [17, 18] and in theta burst stimulation-mediated increases in synaptic pTrkB which are dependent on ERα in females, but not males.

We have contributed to a growing body of literature demonstrating the importance of estrogen receptor signaling in response to cerebral ischemia [7, 19–21]. Neuroprotective effects of estradiol in ovariectomized adult female rodents following MCAO are found to be dependent on ERα but not on ERβ [22]. Additionally, ERα is differentially increased in adult females compared to adult males in ischemic cortical brain regions after MCAO [23], and selective deletion of neuronal ERα also prevents estradiol dependent neuroprotection after MCAO [24]. In addition to adult models of ischemia, other investigators have shown that ERα plays a role in neuroprotection during the neonatal period. Ircariin, which is a flavonoid glycoside, exerts a neuroprotective effect both in P7 mice following the Vannucci’s model of HI and in a mouse hippocampal neuronal cell line (HT22) during OGD by activating ERα and reducing apoptosis [25]. When P6 rat pups or the immature oligodendroglia cell line (OLN-93) is exposed to hypoxia estradiol prevents neonatal oxygen-induced white matter injury [26]. It has been suggested that rather than a ligand-based concentration effect, differential ERα nongenomic signaling appears to play the most important role in mediating neuroprotective actions of estradiol in cerebral ischemia [21]. However, the mechanisms by which ERα activation confers neuroprotection after cerebral ischemia in neonatal brains remains unclear.

We recently demonstrated that in vivo HI induces a sex-specific increase in TrkB phosphorylation in female, but not male neonate hippocampi [7, 8]. Additionally, treatment with 7,8-DHF enhances this HI-induced hippocampal TrkB phosphorylation [7], and results in a profound improvement in hippocampal neuronal survival only in females [8]. We went on to demonstrate that female-specific TrkB phosphorylation and decreased apoptosis after 7,8-DHF treatment was eliminated in ERα null mutant mice [7]. Thus, the female neuroprotective phenotype following HI is linked to TrkB phosphorylation and is dependent upon ERα expression.

Hormonal environment during the perinatal period is associated with the organization of male and female neonatal brains and sex-specific behavioral phenotypes [27]. In male mice brains there is a testosterone (T) surge immediately following birth which reaches levels like those seen in adolescent male mice. The T surge is resolved by four hours post-birth and returns to the levels seen in females [28]. The perinatal T surge seen in males is linked to masculinization of neuronal morphology and behavior [29] and the development of some neuronal circuitries that support sex-specific behaviors and physiological functions in adulthood [30]. Interestingly, systemic priming of female pups by T prior to culturing diminishes female hippocampal neurons phenotypical neuroprotection during hypoxia [31]. However, the relationship between the perinatal increase in T and the sexually differential expression of ERα in a stress condition such as neonatal HI has not been studied.

In this study, we subjected sexed hippocampal neurons to oxygen glucose deprivation and reoxygenation (OGD/REOX) to study the sex differences in TrkB mediated ERα dependent neuroprotection. While it is difficult to successfully model HIE in a single in vitro system, OGD has been widely used to investigate biochemical and molecular mechanisms that lead to post-HI injury [32]. Our results demonstrate that the female- specific neuroprotective mechanisms previously shown to operate in vivo [7] are autonomous functions of female hippocampal neurons *in vitro.* Thus, ERα expression is increased in female hippocampal neurons following in vitro ischemia and is required for TrkB mediated neuroprotection. In addition, we show that T treated female hippocampal neurons fail to up-regulate ERα expression following OGD, resulting in reduction of neuroprotection to the levels seen in male hippocampal neurons. These results point to a model of an intrinsic neuroprotective pathway in female hippocampal neurons that involves ERα-dependent activation by 7,8-DHF and subsequent TrkB phosphorylation. This pathway is rendered unresponsive by pre-exposure to androgens through mechanisms that remained to be determined.

## Methods

### Materials

Anti-mouse monoclonal anti-microtubule associated protein 2 (MAP-2), goat serum, propidium iodide (PI), cytosine beta-d-arabinofuranoside (AraC), bovine serum albumin (BSA), triton X, N-[2-[[(Hexahydro-2-oxo-1 H-azepin-3-yl)amino]carbonyl]phenyl]-benzo[b]thiophene-2-carboxamide (ANA-12), tyramine, testosterone (T), and 7,8-DHF were from Sigma (St. Louis, MO). Rabbit polyclonal anti-phospho-TrkB (Tyr705) was from Signalway Antibody (College Park, MD) which was validated previously [7]. Rabbit polyclonal anti-ERα was from EMD/Millipore (Temecula, CA). Goat anti-rabbit Alexa Fluor 488-conjugated IgG and goat anti-mouse Alexa Fluor 546-conjugated IgG, Streptavidin Alexa fluor 488 conjugate, PlatinumTaq Master Mix, trypsin-EDTA, HBSS, penicillin/streptomycin, neurobasal® medium, B-27® medium, Taqman probes for ERα, GAPDH, and SuperScript® VILO™ cDNA synthesis kit was from Life Technologies (Carlsbad, CA). Thermo-Scientific™ EZ-Link™ Sulfo-NHS-LC-Biotin and Labeling Kit and Hoechst 33342 was from Thermo-Fisher (Waltham, MA). ABS Peroxidase Elite kite and Vectashield mounting media with DAPI was from Vector Laboratories (Burlingame, CA). Bullseye taqprobe Master Mix was from Midsci (St. Louis, MO).

### Primary hippocampal neuronal culture

Primary hippocampal neuronal cultures were prepared as described previously with some modifications([33, [34); either from individual P1 old C57BL/6 J mouse pups in the 7,8-DHF dose response experiments at DIV 2 or by pooling hippocampi from same sex P1 pups in the cell survival, IHC and qRTPCR experiments. The cells from the hippocampi were dissociated through enzymatic treatment (0.25% trypsin) and subsequent trituration. They were then seeded on 12-mm-diameter poly-D-lysine/laminin pre-coated coverslips (BD Biosciences, Bedford, MA) at a density of $\sim$ 1 × 10^5^ (one pup per coverslip) for all the experiments except the immunostaining studies, where relatively less dense cultures ($\sim$ 5 × 10^4^ cells per coverslip) was used. The coverslips were placed in 24 well culture dishes containing a serum-free media (neurobasal® media with phenol red plus 2% B27®-nutrient), 0.4 mM glutamine, and 1% penicillin/streptomycin solution. It should be noted that phenol red has been reported to have weak estrogenic action in hippocampal neurons [35]. Initially, to avoid the confounding effects of cytarabine (AraC) treatment and astrocyte contamination, we performed our dose response studies on DIV2 sexed hippocampal neuronal cultures prepared from ERα^+/+^ mice hippocampi. However, in the OGD/REOX studies we used DIV7 hippocampal neurons that had been treated on DIV3 with the mitotic inhibitor AraC (5 µM) (36) for 24 h. AraC is an established method of inhibiting glial cell proliferation in neuronal cultures [37]. It is reported that the use of AraC (5 µM) along with serum-free maintenance medium in neuronal culture is not conducive to the growth and survival of astrocytes, thereby resulting in the predominant neuronal population with only 6–8% astrocyte contamination [34]. This allowed us to use cultures that were enriched for neurons and had reached stage 4 maturity (polarized morphology) [38].

### ERα^−/−^ knockout mice

ER**α** heterozygous C57BL/6 mice (ERα^+/−^) were bred and sexed hippocampi from all the pups born were cultured on an individual coverslip at P1. At the same time the toe clippings were taken for genotyping. Only the coverslips that have ERα^−/−^ hippocampal neurons were used for the ERα^−/−^ experiments. Genotypes were determined by polymerase chain reaction (PCR) of genomic DNA from finger or toe clippings: clippings were heated at 95 °C for 45 min in 50 mM NaOH and neutralized with equal volume of 1 M Tris buffer, pH 6.8. 1 µL of this DNA solution was added to 19 µL of the following: 0.25 mM of primers for the ERα gene, 1X GoTaq Buffer (Promega, Madison, WI), 0.2 mM each deoxynucleotide (Promega, Madison, WI) and 8 U Platinum Taq. PCR was performed on a BioRad T100 cycler for 30 cycles as follows: 95 °C for 3 min, denaturation at 95 °C for 30 s, annealing at 58 °C for 30 s (ERKO PCR1) or 51 °C for 30 s (ERKO PCR2), and elongation at 72 °C for 1 min. PCR products were separated electrophoretically on an ethidium bromide-containing 2% agarose gel and visualized under UV illumination as described before [7].

### Preparation of sexed hippocampal neuronal cultures

It has been reported that mouse sex can be determined at P1-3 by looking for the presence of a small dark spot between the anogenital opening which is present only in males [39]. To confirm this, sex genotypes were determined by PCR of DNA from tail biopsies in P0 to P1 pups [39]. Biopsy samples are boiled at 95 °C for 1 h in 50 mM NaOH and neutralized with equal volume of 1 M Tris buffer, pH 6.8. 20 ng/ml DNA were added to 1 pmol of primers for the Y-chromosome specific mRNASYR: and the X-chromosome mRNAMYOG [39] and Platinum Taq Master Mix. PCR was performed on a BioRad T100 cycler as follows: 95 °C for 3 min, denaturation at 95 °C for 30 s, annealing at 58 °C for 30 s (PCR1) or 51 °C for 30 s (PCR2), and elongation at 72 °C for 1 min. PCR were performed for 30 cycles. PCR products were separated electrophoretically on a 2% agarose gel and visualized with ethidium bromide. In all mice that we tested the sex genotyping correlated with the visual sexing (results not shown).

### 7,8-DHF dose response

DIV2 sexed hippocampal neurons grown on coverslips were exposed to various doses of 7,8-DHF (0.2, 1.0, 3.0, 30, and 100 µM) or vehicle control (0.001% DMSO in culture media) for 15 min. Coverslips were then quickly rinsed with 0.1 M TBS, fixed and stained for MAP-2 and p-TrkB as described below. A dose response curve that included both male and female neurons was generated and the dose that increased phosphorylation by $\sim$ 70% was used for subsequent experiments.

### Oxygen glucose deprivation (OGD)/ reoxygenation (REOX)

Hippocampal neurons grown on coverslips at DIV7 were placed in the well of a 24 well culture plate, rinsed with OGD-MEM solution (in mM: Na^+^ 130, K^+^ 5.4, CaCl_2_ 1.3, MgSO_4_ 0.8, KH_2_PO_4_ 0.4, Na_2_HPO_4_ 0.3, NaHCO_3_ 4, HEPES 20), and then covered with 0.2 ml OGD-MEM. The plate was then placed in an OGD incubator (1% O_2_, 5% CO_2_, balance N_2_, 37ºC) on an orbital shaker (55 rpm) which was timed to rotate for the first 30 min of OGD incubation. After four hours of OGD incubation, the plate was removed, the OGD-MEM replaced with neuronal media, and the plate returned to a normoxic incubator (5% CO_2_, 37ºC) for 3 h REOX in all mRNA expression experiments and 24 h REOX for all immunocytochemistry experiments. Cells were treated with either media containing 3 µM 7,8-DHF and/or ANA-12 (TrkB antagonist, 100 µM) during REOX.

### Immunostaining - pTrkB ^Y705^ and MAP2

In both the dose response studies and following 4 h OGD and 24 h REOX, coverslips were quickly rinsed with 0.1 M TBS and then fixed with 4% paraformaldehyde for 15 min at room temperature. Each coverslip is considered as *n* = 1. Fixed coverslips were rinsed with 0.1 M TBS 6 × 2 min, blocked with TBS^++^ (10% goat serum, 1% BSA & 0.1%Triton X in 0.1 M TBS), incubated with anti-p-TrkB^Y705^ (1:100) and anti-MAP-2 (1:500) in TBS^++^ for 1 h at 37° C and then overnight at 4° C. After rinsing 3 × 2 min with 0.1 M TBS, coverslips were incubated in Alexa-Fluor 488 anti-rabbit goat and Alexa-Fluor 556 anti-mouse goat diluted in TBS^++^ for 1 h at 37 °C. After rinsing 3 × 2 min with 0.1 M TBS, coverslips were mounted on slides using Vectashield hard set with DAPI (Vector Labs, Burlingame, CA).

### Immunostaining for ERα and MAP2

ERα was detected in hippocampal cultures using an avidin-biotin complex kit coupled to tyramine-amplification with detection via streptavidin Alexa Flour 488. Briefly, coverslips were quickly rinsed with 0.1 M TBS and then fixed with 4% paraformaldehyde for 15 min at RT. Coverslips were then washed 10 × (1 h total) with KPBS (this protocol was used for all subsequent washes) and then incubated with anti-ERα (1:50) in KPBS for 1 h at RT and then at 4 °C for 48 h. Using a Vectastain ABC kit, coverslips were washed followed by incubation with biotinylated secondary antibody (Goat, anti-rabbit), washed again and then incubated with an avidin biotinylated enzyme complex as per manufacturer’s instructions. Next, coverslips were washed and then incubated with freshly prepared biotinylated tyramine [40] for 20 min at room temperature. After washing the coverslips were then incubated with streptavidin Alexa-Flour-488 (1:2000) for 1 h at RT and then overnight at 4 °C. The following day the coverslips were washed and then counterstained with anti-MAP-2 (1:500) and Alexa-Fluor 556 as described above.

### Microscopy and image analysis

All the imaging was done on Nikon A1R-Si Laser Scanning Confocal microscope using a 20X objective. In the dose response studies, because of variable cell density at DIV2, as few as three and as many as nine fields of fluorescent images were collected while for the OGD/REOX studies at DIV 7, six random fields of fluorescent images per coverslip were collected. Then each field was sampled for DAPI (405 nm ex. / 450 nm em.), p-TrkB (488 nm ex. / 515 nm em.), and MAP-2 (546 nm ex./ 595 nm em.) per coverslip. Using ImageJ [41 imaging processing software, the MAP-2 image was opened and the auto threshold function used to filter for MAP-2 positive areas. The create selection function was followed by the restore selection function to transfer the MAP-2 positive area to the corresponding p-TrkB image. For both DIV2 and DIV7 experiments, the mean intensity of the p-TrkB within the MAP-2 positive area was recorded along with the background intensity. The background intensity values in arbitrary units (A.U.) were subtracted from mean intensity values for all the fields in the coverslip and which were then averaged (each coverslip is considered *n* = 1). All image files names were coded to blind them to the experimenter. Coverslips stained for ERα were imaged for DAPI (405 nm ex. / 450 nm em.), ERα (488 nm ex. / 515 nm em.), and MAP-2 (546 nm ex./ 595 nm em.).

### Neuronal survival

Following 4 h OGD and 24 h REOX, coverslips were moved to glass-bottomed 35 mm culture dishes and rinsed with HEPES-MEM (OGD-MEM with the addition of 5 mM glucose). A loading solution containing 0.2 ug/ml PI and 10 ug/ml Hoechst 33342 in HEPES-MEM was added. Coverslips were incubated at 37ºC for 30 min. The culture dish was then rinsed with HEPES-MEM and placed on the stage of Nikon A1 confocal and images of 6 fields (20x) were collected to quantify total cells (Hoechst 33342 positive, 405 nm ex./ 450 nm em.) and dead cells (PI positive, 546 nm ex./ 595 nm em.). The PI and Hoechst 33342 images were opened in Image J, an auto threshold applied and the analyze particles function used to count the number (10–100 sq pixel size) of PI positive and Hoechst 33342 positive nuclei. The PI/Hoechst 33342 ratio was subtracted from one to yield the percentage of surviving cells. The percent surviving cells for the six fields per coverslip were then averaged (*n* = 1). All image files names were coded to blind them to the experimenter.

### Quantitative polymerase chain reaction (96-well qRTPCR)

Following 4 h OGD and 3 h REOX, RNA was extracted from hippocampal neurons using a RNeasy® mini kit (Qiagen, Hilden, Germany) according to manufacturer’s instructions. Briefly, coverslips were rinsed quickly with HBSS and the cells lysed, scrapped, triturated, and transferred to a spin column where RNA binds to the silica membrane. After washing to remove contaminates, RNA was extracted from the column with 30 µl of water. The amount of total RNA was determined from the optical density measurements at 260 and 280 nm (NanoDrop 2000c, Thermo Scientific, Wilmington, DE). Reverse transcription was performed using the reagents and protocols from a SuperScript® VILO™ cDNA kit and 0.5 µg of total RNA. For quantitative PCR amplification 16.66 ng of cDNA was used. Each reaction (final volume 15–20 µl) for a single mRNA was done in duplicate and consisted of predesigned gene-specific primers and probes for ERα (Mn00433149_m1), GAPDH (Mm99999915_g1) and bullseye taqprobe Master Mix on a 96 well plate. PCR amplification was accomplished using an Applied Biosystem 7500 qPCR system (Life Technologies, Carlsbad, CA) running a standard amplification protocol (50° C 2 min, 95° C 10 min, 95° C 15 s, 60° C 1 min, 40 cycles). Cycles to threshold values were analyzed using the System 7500 SDS software (Life Technologies, Carlsbad, CA). Relative mRNA expression was calculated by the 2^− ΔΔCT^ method using GAPDH as an internal control and a pooled hypothalamus sample as a cross plate control. Results were expressed relative to male normoxic neurons.

### Quantitative polymerase chain reaction (384 well-qRTPCR)

For experiments investigating ERα mRNA expressions following OGD/REOX with and without T treatment a 384-well RT-qPCR was used. Briefly, RNA was extracted from tissues using Qiagen RNeasy mini-spin kit (Qiagen, Germantown, MD) according to manufacturer’s instructions. RNA concentration and purity were determined using a NanoDrop 2000c. The cDNA was synthesized from 1000 ng total RNA using a superscript Vilo synthesis kit (Life Technologies, Carlsbad, CA) using manufacturer’s instructions. RT-qPCR was performed using TaqMan mRNA Expression Assay probes (ERα, Mn00433149_m1; GAPDH, Mm99999915_g1, Life Technologies, Carlsbad, CA). A master mix solution was made consisting of 0.5 µl TaqMan mRNAexpression assay probe (20X), 7.5 µl TaqMan mRNAexpression master mix, and 5.0 µl nuclease free water. Samples are loaded in duplicate on a 384 –well plate with a final reaction volume of 15 µl, consisting of 2.0 µl of cDNA (16.66 ng) and 13.0 µl of master mix solution. The plate was sealed with a plate sealer and briefly centrifuged (1000 rpm, 2 min) and the qPCR was performed using the ViiA-7 Real Time PCR System (Applied Biosystems Waltham, MA) following the manufacturer’s instructions. Following the completion of the RT-qPCR run, relative mRNA expression levels were calculated using the 2^− ΔΔCT^ method upon normalization to the expression levels of GAPDH reference gene.

### Pretreatment with testosterone

Primary hippocampal cultures derived from 1-day old C57BL/6 mouse pups were treated daily with 10 nM of T. T treatment was started on DIV2 and maintained until DIV7. For treatments, a stock solution of T (10 µM) was made in 100% ethanol. On each day of drug treatment, stock T or vehicle (100% ethanol) were diluted (1:1000) and added directly to the neurobasal media resulting in a final T concentration of 10 nM.

### Statistical analysis

All statistical analyses were conducted using SAS software (SAS Institute Inc., Cary NC), version 9.4. The E_50_ for 7,8-DHF was calculated by fitting the dose response data with a 3-parameter E_max_ model as follows:

$$\text{p}\text{T}\text{r}\text{k}\text{B} \text{i}\text{n}\text{t}\text{e}\text{n}\text{s}\text{i}\text{t}\text{y}={\text{E}}_{\text{m}\text{a}\text{x}}+\frac{{\text{E}}_{\text{m}\text{a}\text{x}}-{\text{E}}_{\text{m}\text{i}\text{n}}}{1+{\left(\frac{\text{d}\text{o}\text{s}\text{e}}{{\text{E}}_{50}}\right)}^{{\varnothing}}}$$

where Emax, Emin, and E50 denote the dose levels of 7,8-DHF at which the maximum, minimum and 50% intensity of p-TrkB immunostaining is achieved, and ∅ denotes the slope parameter of the dose-response curve. The maximum likelihood method was used to estimate the parameters of the Emax model. The E50 was calculated and reported along with the corresponding 95% confidence intervals (CIs) for both males and females separately and males and females combined. Relative mRNAexpression and p-TrkB fluorescence intensity levels were summarized in terms of means ± standard errors, stratified by experimental condition. In order to evaluate the effect of in vitro and in vitro ischemia and 7,8-DHF treatment on the p-TrkB immunoexpression in sexed hippocampal neurons, multi-factorial analysis of variance (ANOVA) was conducted. In this analysis treatment, genotype and sex were included as main factors. Two and three-way interaction effects were included and evaluated. Sex specific differences between treatment groups were evaluated by evaluating sliced (by sex) three-way interaction contrasts. Analogously, Analogously, multi-factorial ANOVA with treatment and genotype as main factors was performed to evaluate 7,8-DHF-mediated neuronal survival was dependent on ERα in cultured hippocampal neurons. A two-way ANOVA with OGD and REOX exposure as main factors was used to evaluate ERα mRNA expression in hippocampal neurons following OGD/REOX. Model assumptions were examined to verify model assumptions. All reported p-values are two-sided and *P* < 0.05 was used to define statistical significance.

## Results

### Female ERα^+/+^ hippocampal neurons elicited higher pTrkB immunoexpressions under normoxic conditions and this response was ERα dependent

**Fig. 1:**
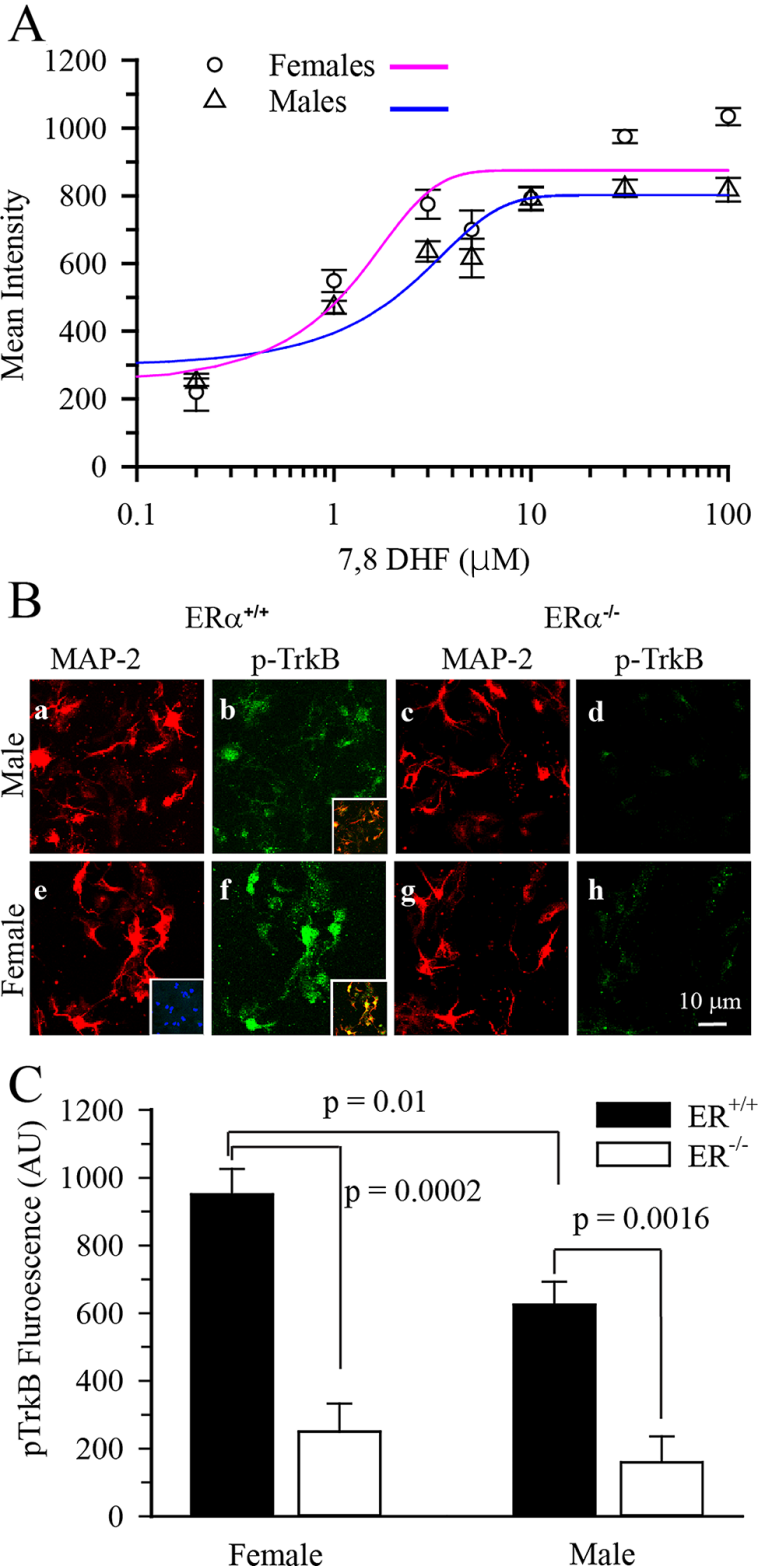
Cultured hippocampal neurons had a sexually differential response to 7,8-DHF-mediated p-TrkB receptor activation that was ERα dependent DIV2 hippocampal neuronal cultures were subjected to various levels of 7,8-DHF for 15 min and the cells fixed and immunostained for p-TrkB and MAP-2. **A.** Summary figure of p-TrkB fluorescence intensity in MAP-2 positive cells determined in sexed hippocampal neurons cultured from ERα^+/+^ mice and subjected to various concentrations of 7,8-DHF for 15 min. Data are mean ± SEM, *n* = 16–27. Points were fitted with a three-parameter sigmoidal function. **B**. Representative images of DIV2 cultured ERα^+/+^ and ERα^-/-^ hippocampal neurons immunostained with MAP-2(red) and p-TrkB (green) 15 min post-application of 3 µM 7,8-DHF. Inset **(b, f)**: overlay of MAP-2 and p-TrkB images. Inset (d): primary antibody control (p-TrkB and MAP-2). **C**. Summary figure of p-TrkB fluorescence intensity in MAP-2 positive ERα^+/+^ and ERα^-/-^ hippocampal neurons 15 min post-application of 3 µM 7,8-DHF. Data are mean ± SEM adjusted by vehicle control, ERα^+/+^, *n* = 4, ERα^-/-^*n* = 10–12

In order to determine the optimum dose of the 7,8-DHF in hippocampal neurons in culture we conducted a dose-response experiment. Sexed primary hippocampal neurons in culture prepared from ERα^+/+^ mice were treated with various concentrations of 7,8-DHF at DIV2. Then we constructed dose response curves and calculated the E50 for the intensity of p-TrkB immunostaining in response to various doses of 7,8-DHF for male and female primary hippocampal neurons as described under methods. There was no significant difference between the E_50_ of male (1.81 µM 7,8-DHF) and female (2.58 µM 7,8-DHF) (*p* = 0.50) hippocampal neurons (Fig. 1A). In a fit model of the combined p-TrkB intensity response in female and male hippocampal neurons, 3 µM 7,8-DHF resulted in a response that was 71% of the E_max_. Therefore, treatment with 3 µM 7,8-DHF was used in in the remaining studies. When hippocampal neurons were treated with 3 µM 7,8-DHF for 15 min there was statistically stronger staining response in ERα^+/+^ female hippocampal neurons (951 ± 75 A.U.) compared to male neurons (625 ± 68 A.U.) (Fig. 1B, C, *p* = 0.01). This sexually differentiated response to 3 µM of 7,8-DHF was abolished in ERα^−/−^ hippocampal neurons (*p* = 0.0002, Fig. 1C).

Cultured hippocampal neurons had a sexually differential response to 7,8-DHF-mediated p-TrkB receptor activation that was ERα dependent. DIV2 hippocampal neuronal cultures were subjected to various levels of 7,8-DHF for 15 min and the cells fixed and immunostained for p-TrkB and MAP-2. **A.** Summary figure of p-TrkB fluorescence intensity in MAP-2 positive cells determined in sexed hippocampal neurons cultured from ERα^+/+^ mice and subjected to various concentrations of 7,8-DHF for 15 min. Data are mean ± SEM, *n* = 16–27. Points were fitted with a three-parameter sigmoidal function. **B**. Representative images of DIV2 cultured ERα^+/+^ and ERα^-/-^ hippocampal neurons immunostained with MAP-2(red) and p-TrkB (green) 15 min post-application of 3 µM 7,8-DHF. Inset **(b, f)**: overlay of MAP-2 and p-TrkB images. Inset **(d)**: primary antibody control (p-TrkB and MAP-2). **C**. Summary figure of p-TrkB fluorescence intensity in MAP-2 positive ERα^+/+^ and ERα^-/-^ hippocampal neurons 15 min post-application of 3 µM 7,8-DHF. Data are mean ± SEM adjusted by vehicle control, ERα^+/+^, *n* = 4, ERα^-/-^*n* = 10–12

### Female ERα^+/+^ hippocampal neurons elicited higher pTrkB immunoexpressions following in vitro ischemia which was enhanced with 7,8-DHF treatment

**Fig. 2:**
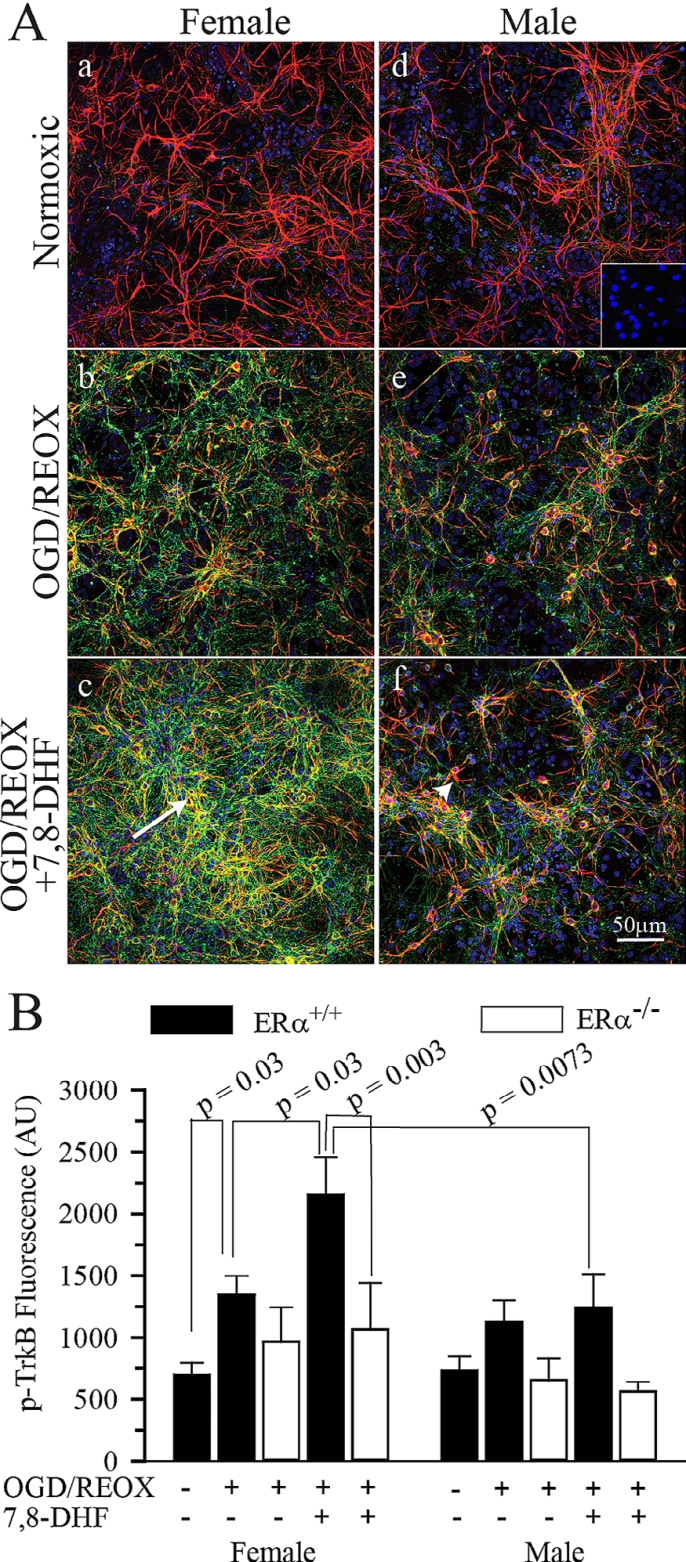
p-TrkB immunoactivity increased following OGD/REOX .ERα^+/+^ and ERα^−/−^ hippocampal neurons were grown on coverslips and subjected to 4 h OGD followed by VC or 3 µM 7,8-DHF during 24 h REOX. Cells were fixed and stained with anti-p-TrkB and anti-MAP-2 antibodies. **A**. Representative images of male and female ERα^+/+^ hippocampal neurons under normoxic conditions or after 4 h OGD followed by 24 h REOX with and without 7,8-DHF treatment (3µM). OGD/REOX resulted in increased p-TrkB staining in male and female hippocampal neurons. However, 7,8-DHF treatment resulted in a further increase in p-TrkB staining in female neurons, but not males. Inset: primary antibody control (p-TrkB and MAP-2). Arrow = co-localized p-TrkB and MAP-2 staining; Arrowhead = lack of p-TrkB staining. **B**. Summary figure of p-TrkB staining intensity in ERα^+/+^ and ERα^−/−^ hippocampal neurons grown on coverslips and subjected to 4 h OGD followed by 24 h REOX with and without 7,8-DHF treatment (3µM). Data are mean ± SEM, ERα^+/+^, *n* = 4–11; ERα^−/−^, *n* = 6. Significance was determined by multi-factorial analysis of variance

Next, we investigated the effect of in vitro ischemia and 7,8-DHF treatment on the p-TrkB immunoexpression in sexed hippocampal neurons. Sexed hippocampal neurons grown on coverslips were subjected to 4 h of OGD followed by VC or 3 µM 7,8-DHF during 24 h of REOX. The coverslips were then fixed and stained for p-TrkB and MAP-2 immunoexpressions. As seen in Fig. 2A, under normoxic conditions p-TrkB staining was weak in both male and female hippocampal ERα^+/+^ neurons. However, following OGD/REOX p-TrkB immunostaining increased in MAP-2 positive male and female ERα^+/+^ hippocampal neurons. There was also an increase in p-TrkB staining in non-MAP-2 positive areas of the cultures. At DIV7, MAP-2 staining has been reported to be almost exclusively dendritic so these non-MAP-2 staining areas most likely represent staining within the network of axonal processes [42]. However, we cannot rule out a negligible MAP-2 negative p-TrkB staining from non-neuronal cells present our culture. Interestingly, with 3 µM 7,8-DHF treatment there was a further enhancement in p-TrkB staining in both MAP-2 positive and MAP-2 negative areas in female hippocampal ERα^+/+^ neurons. This increase was absent in male ERα^+/+^ cultures (Fig. 2A). Figure 2B summaries the p-TrkB staining intensities following OGD/REOX in ERα^+/+^ and ERα^−/−^ hippocampal cultures. When compared to normoxic hippocampal neurons within their sex, OGD/REOX resulted in a 1.93 ± 0.22-fold increase in p-TrkB staining in ERα^+/+^ female hippocampal neurons and a 1.82 ± 0.21-fold increase in male ERα^+/+^ hippocampal neurons. In ERα^−/−^ hippocampal neurons, there was a smaller increase in p-TrkB staining of 1.05 ± 0.26-fold in females and 0.85 ± 0.29-fold in males (Fig. 2B). When hippocampal neurons are treated with 3 µM 7,8-DHF during OGD/REOX, p-TrkB staining increased further to 3.09 ± 0.43-fold in female ERα^+/+^ hippocampal neurons. This post-OGD 7,8-DHF induced increase in p-TrkB staining was absent in male ERα^+/+^ hippocampal neurons and in cultures from either male or female ERα^−/−^ mice (Fig. 2B). Thus, the increases in phosphorylation of TrkB following OGD/REOX were dependent on the presence of ERα. In addition, there was a sexually differentiated response to the 7,8-DHF induced phosphorylation of TrkB following OGD/REOX in ERα^+/+^ hippocampal neurons.

p-TrkB immunoactivity increased following OGD/REOX ERα^+/+^ and ERα^−/−^ hippocampal neurons were grown on coverslips and subjected to 4 h OGD followed by VC or 3 µM 7,8-DHF during 24 h REOX. Cells were fixed and stained with anti-p-TrkB and anti-MAP-2 antibodies. **A**. Representative images of male and female ERα^+/+^ hippocampal neurons under normoxic conditions or after 4 h OGD followed by 24 h REOX with and without 7,8-DHF treatment (3µM). OGD/REOX resulted in increased p-TrkB staining in male and female hippocampal neurons. However, 7,8-DHF treatment resulted in a further increase in p-TrkB staining in female neurons, but not males. Inset: primary antibody control (p-TrkB and MAP-2). Arrow = co-localized p-TrkB and MAP-2 staining; Arrowhead = lack of p-TrkB staining. **B**. Summary figure of p-TrkB staining intensity in ERα^+/+^ and ERα^−/−^ hippocampal neurons grown on coverslips and subjected to 4 h OGD followed by 24 h REOX with and without 7,8-DHF treatment (3µM). Data are mean ± SEM, ERα^+/+^, *n* = 4–11; ERα^−/−^, *n* = 6. Significance was determined by multi-factorial analysis of variance

### 7,8-DHF-mediated neuronal survival was dependent on ERα in cultured hippocampal neurons

**Fig. 3:**
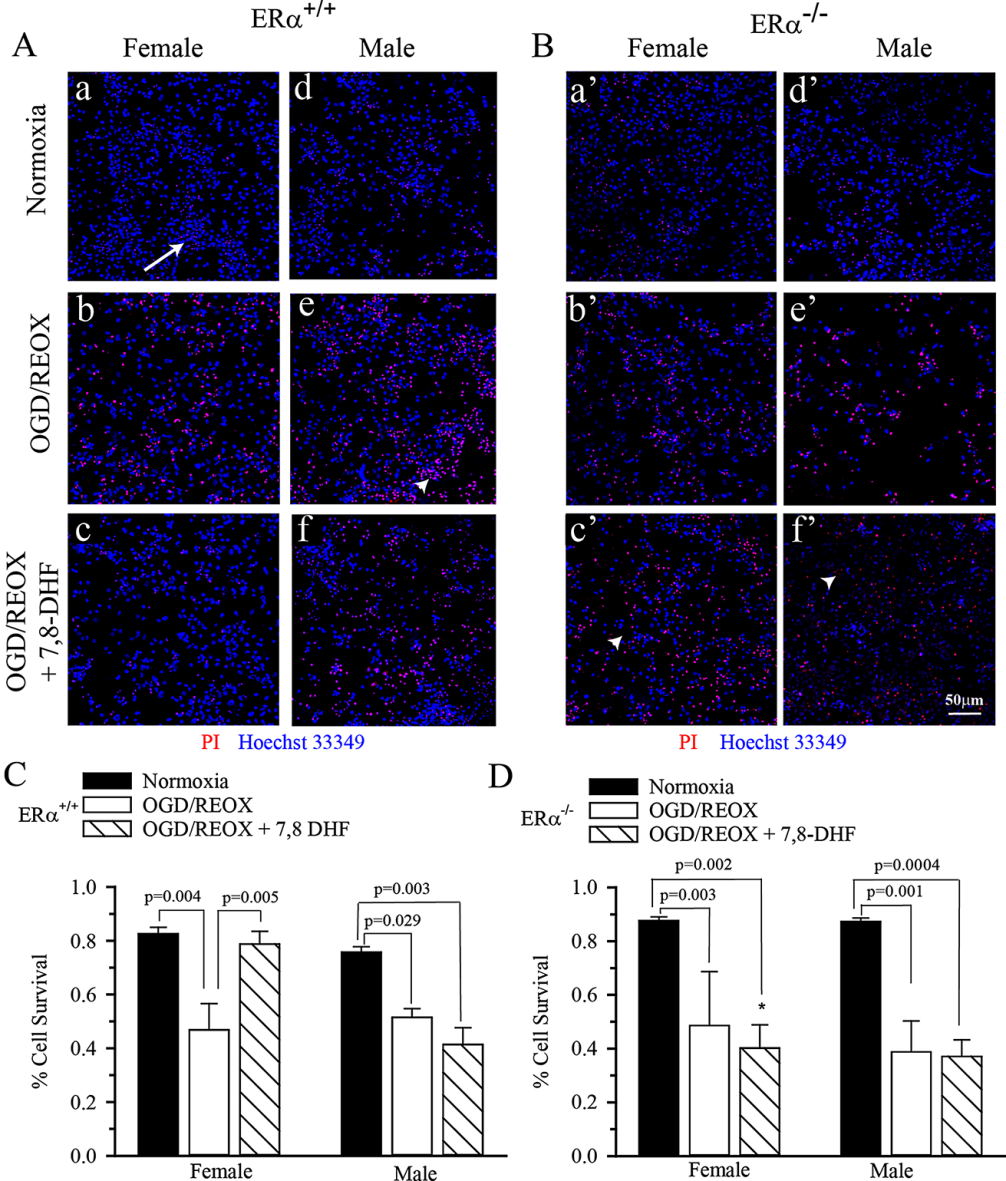
7,8-DHF-mediated neuroprotection was dependent on ERα in cultured hippocampal neurons. ERα^+/+^ and ERα^−/−^ hippocampal neurons were grown on coverslips and subjected to 4 h OGD followed by VC or 3 µM 7,8-DHF during 24 h REOX. Cells were stained with Hoechst 33349 and PI to detect total and dead cells respectively. **A.** Representative images of male and female ERα^+/+^ hippocampal neurons under normoxic conditions or after 4 h OGD followed by 24 h REOX with and without 3 µM 7,8-DHF treatment. Arrow **=** Hoechst 33349 nuclear staining (all cells); Arrowhead **=** co-localized Hoechst 33349 and PI (dead cells) **B.** Representative images of male and female ERα^−/−^ hippocampal under normoxic conditions or after 4 h OGD followed by 24 h REOX with and without 3 µM 7,8-DHF treatment. **C.** Summary figure of % cell survival in ERα^+/+^ hippocampal neurons subjected to 4 h OGD and 24 h REOX. OGD resulted in a decrease in cell survival in male and female ERα^+/+^ hippocampal neurons. Treatment with 3 µM 7,8-DHF rescued female ERα^+/+^ hippocampal neurons, but not male neurons. Data are mean ± SEM, *n* = 5–7. **D**. Summary figure of % cell survival in ERα^−/−^ hippocampal neurons subjected to 4 h OGD and 24 h REOX. OGD resulted in a decrease in cell survival in male and female ERα^−/−^ hippocampal neurons. However, treatment with 3 µM 7,8-DHF failed to rescue either male or female ERα^−/−^ hippocampal neurons. Data are mean ± SEM, *n* = 3–6. * *p* = 0.004 vs. female ERα^+/+^ OGD/REOX + 7,8-DHF. Significance was determined by multi-factorial analysis of variance

Increases in TrkB phosphorylation post-OGD/REOX could result in up-regulation of signaling pathways that promote cell survival. To test this, we determined cell survival following 4 h OGD and 24 h REOX (Fig. 3A,B). Under normoxic conditions the % cell survival was not different between male and female neurons or between ERα^+/+^ and ERα^−/−^ (average in all groups, 0.83 ± 0.02%). 4 h of OGD plus 24 h of REOX resulted in a dramatic decrease in % cell survival in all groups that was not significantly different between sexes or genotypes (average of all groups, $\sim$ 56% decrease, Fig. 3C,D). However, when 3 µM 7,8-DHF was applied during REOX in female ERα^+/+^ hippocampal neurons the % cell survival was rescued (0.83 ± 0.02 vs. 0.79 ± 0.04, normoxic vs. treated female, Fig. 3C,D). 7,8-DHF treatment failed to increase % cell survival in male ERα^+/+^ neurons or ERα^−/−^ neurons of either sex. Thus, activation of TrkB with its agonist resulted in protection in female but not male hippocampal neurons in an ERα-dependent manner.

### ERα mRNA expression in hippocampal neurons following OGD/REOX

**Fig. 4:**
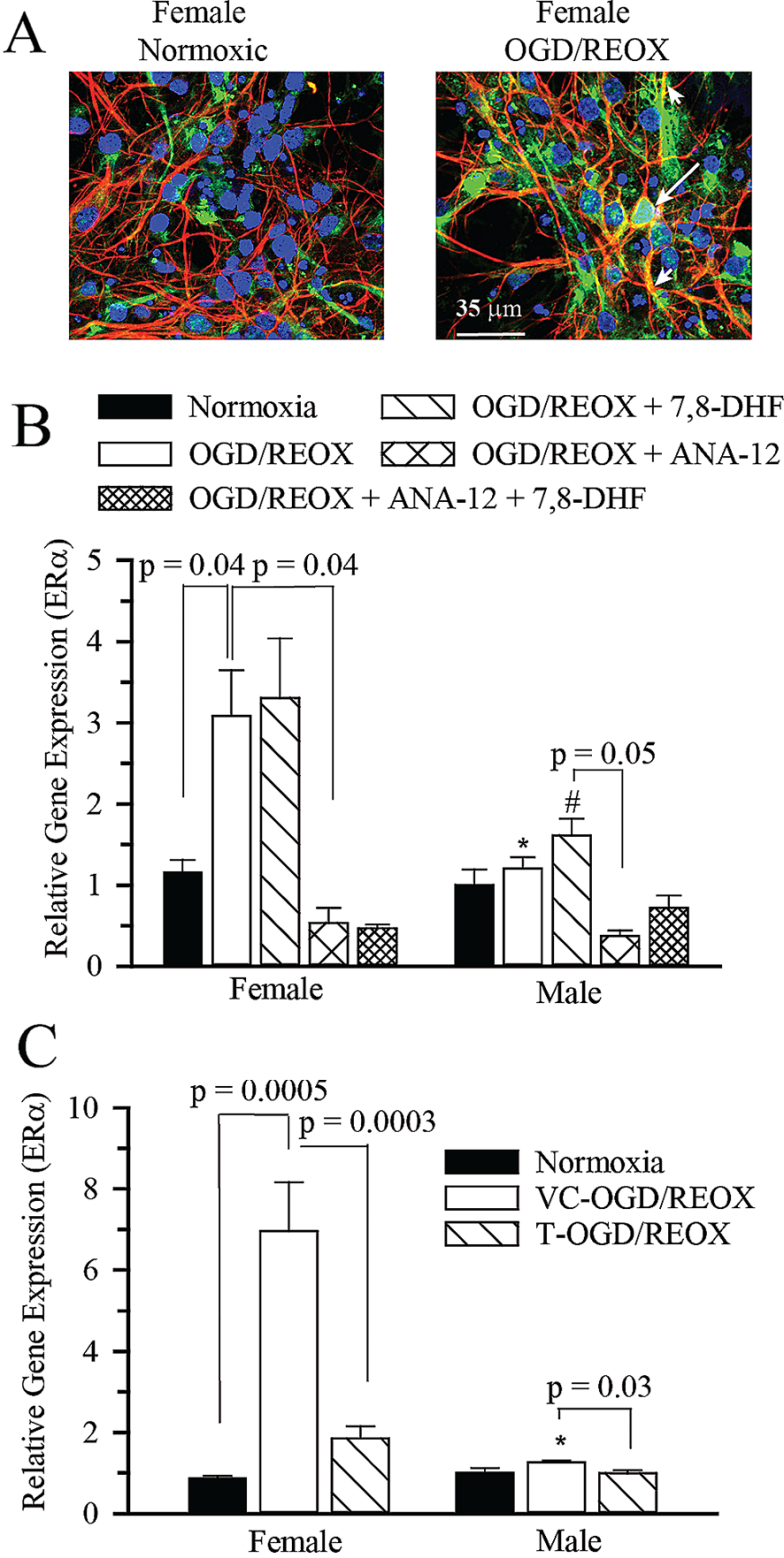
ERα mRNA expression increased in female hippocampal neurons following OGD/REOX and was blocked by ANA-12 treatment or T pretreatment. At the end of REOX, cells were either stained for ERα or harvested for mRNA extraction and probed for mRNAexpression of ERα. **A**. Representative images of female hippocampal neurons stained for ERα (green) and counterstained with MAP-2 (red) and DAPI (blue) after 4 h OGD and 24 h REOX. Arrow: ERα nuclear staining. Arrowhead: ERα neurite staining. **B**. Summary figure of relative ERα mRNAexpression in cultured hippocampal neurons under normoxic conditions and after exposure to 4 h OGD and 3 h REOX. Cells were treated with either vehicle control, 3 µM 7,8-DHF, 100 µM ANA-12 or 3 µM 7,8-DHF + 100 µM ANA-12 during REOX. Values are expressed relative to male normoxia. Values are mean ± SEM, *n* = 3–12. * *p* = 0.009 vs. female 4 h OGD and 3 h REOX; # *p* = 0.02 vs. female 4 h OGD and 3 h REOX + 3µM 7,8-DHF. Significance was determined by multi-factorial analysis of variance. **C.** Relative ERα mRNA expression in cultured hippocampal neurons under normoxic conditions and after 4 h OGD and 3 h REOX. Cells were pretreated with either vehicle control (VC) or 10 nM T (T). Values are expressed relative to male normoxia. Values are mean ± SEM, *n* = 3–8. * *p* = 0.0001 vs. VC treated female 4 h OGD and 3 h REOX. Significance was determined by multi-factorial analysis of variance

Previously, we have reported that ERα mRNA and protein expressions increase in the ipsilateral hippocampi of neonatal mice subjected hypoxia ischemia [7] Initially, we used tyramine amplified ERα immunohistochemical staining to visualize the expression of ERα in female ERα^+/+^ hippocampal neurons. 4 h of OGD and 24 h REOX resulted in an increase in ERα expression both in the nucleus and in the neurites of female hippocampal neurons (Fig. 4A). Then, we investigated the ERα mRNA expressions in sexed hippocampal neurons following 4 h of OGD and 3 h, 6 h, and 24 h of REOX. There was no increase in ERα mRNA expression in neurons at either 6 or 24 h of REOX (data not shown). However, 4 h of OGD and 3 h REOX resulted in a 3.08 ± 0.56 fold increase in ERα mRNA expression in female hippocampal neurons when adjusted to male normoxic values (*p* < 0.04) (Fig. 4B). There was no further effect of 7,8-DHF on ERα mRNA expression in female neurons following 4 h OGD/REOX and 3 h REOX (Fig. 4B). ERα mRNA expression in male neurons did not change following 4 h OGD and 3 h REOX with or without 7,8-DHF treatment. There are studies suggesting that 7,8-DHF may be metabolized to form bioactive products that could result in increases of ERα mRNA expression [43, 44]. To investigate if the increase in ERα mRNA expression in female neurons treated with 7,8-DHF following OGD/REOX was directly related to TrkB activation, we applied 100 µM ANA-12, a TrkB antagonist (Fig. 4B). With ANA-12 application, the OGD/REOX-mediated increase in ERα mRNA expression in female hippocampal neurons was abolished (0.53 ± 0.18, *p* = 0.04); Fig. 4B. Furthermore, the application of 3µM 7,8-DHF did not rescue the ERα expression in ANA-12 treated female neurons (0.47 ± 0.05; Fig. 4B). Compared to female hippocampal neurons, male hippocampal neurons failed to exhibit a significant increase in ERα mRNA expression both after OGD/REOX (*p* = 0.009) or OGD/REOX + 7,8-DHF (*p* = 0.02). Interestingly, ANA-12 treatment in male OGD/REOX hippocampal neurons did result in small but significant decrease in ERα mRNA expression compared to OGD/REOX + 7,8-DHF male hippocampal neurons (*p* = 0.05, Fig. 4B). Taken together, this confirmed that the 7,8-DHF-induced increase in ERα mRNA expression was dependent on TrkB activation.

### Testosterone might modulate the sex differences in TrkB mediated ERα dependent neuroprotection following in vitro ischemia

To investigate the role of the masculinizing effect of T on ERα mRNA expression in sexed hippocampal neurons, we pretreated primary hippocampal neurons daily with 10 nM T from DIV2 through DIV7. We then subjected them to 4 h OGD and 3 h REOX. While OGD/REOX resulted in an increase in ERα mRNA expression in female hippocampal neurons (*p* = 0.0005) compared to normoxia, there was no increase in ERα mRNA expression seen in male hippocampal neurons following OGD/REOX. Interestingly, the T pretreated female hippocampal neurons ERα mRNA expression following OGD/REOX was dramatically decreased compared to nontreated female hippocampal neurons (1.85 ± 0.3 vs. 6.96 ± 1.2, *p* = 0.0003; Fig. 4C) and was not different from female normoxia. In male hippocampal neurons following OGD/REOX, T also resulted in a small but significant decrease in ERα mRNA expression (*p* = 0.03; Fig. 4C).

### 7,8-DHF-mediated female specific neuroprotection was blocked in T pretreated hippocampal neurons following in vitro ischemia

**Fig. 5:**
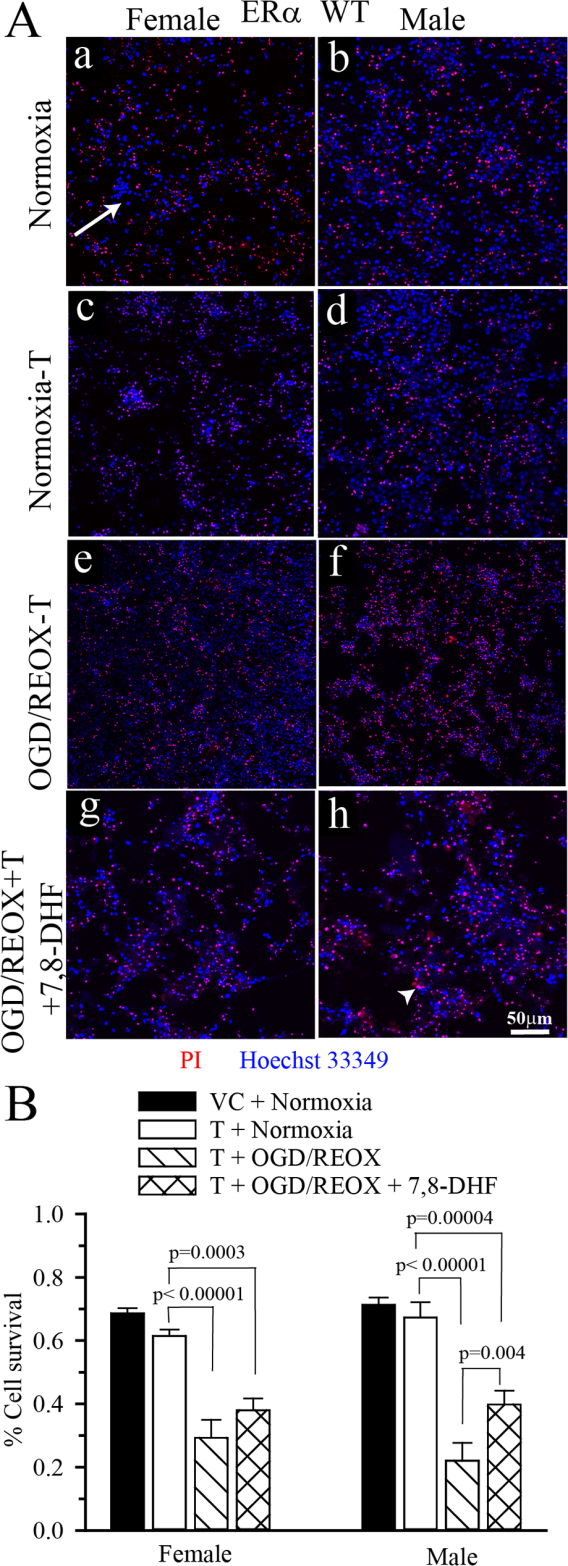
T pretreatment in ERα^+/+^ hippocampal neurons inhibited 7,8-DHF mediated neuroprotection following OGD/REOX ERα^+/+^ and ERα^−/−^ hippocampal neurons pretreated with either VC or 10 nM T were subjected to 4 h OGD followed by VC or 3 µM 7,8-DHF during 24 h REOX. Cells were stained with Hoechst 33349 and PI to detect total and dead respectively. **(A)** Representative images of male and female T or vehicle control pretreated ERα^+/+^ hippocampal neurons under normoxic conditions or after 4 h OGD followed by VC or 3 µM 7,8-DHF during 24 h REOX. Arrow **=** Hoechst 33349 nuclear staining (all cells); Arrowhead **=** co-localized Hoechst 33349 and PI (dead cells) **(B)** Summary figure of % cell survival in vehicle control (VC) and 10 nM T pretreated ERα^+/+^ hippocampal neurons subjected to 4 h OGD and VC or 7,8 µM-DHF during 24 h REOX. OGD resulted in a decrease in cell survival in male and female ERα^+/+^ hippocampal neurons. Following T pretreatment, 7,8-DHF failed to rescue either female ERα^+/+^ hippocampal neurons. Data are mean ± SEM, *n* = 5. Significance was determined by multi-factorial analysis of variance

Next, we investigated whether the T-mediated reduction in ERα mRNA expression observed in female hippocampal neurons would translate into loss of 7,8-DHF-mediated neuroprotection following OGD/REOX. To test this, we determined cell survival following 4 h OGD and 24 h REOX (Fig. 5A,B) in T pretreated hippocampal neurons. Under normoxic conditions, T pretreatment had no effect on the % cell survival in either male and female hippocampal neurons (Fig. 5A,B). When hippocampal neurons were pretreated with T and then subjected to 4 h of OGD plus 24 h of REOX there was dramatic decrease in % cell survival in both sexes; female (0.61 ± 0.02 vs. 0.29 ± 0.06, *p* = 0.0003, Fig. 5B) and male (0.71 ± 0.02 vs. 0.22 ± 0.06, *p* < 0.00001, Fig. 5B). In Fig. 3C, we showed that application of 3µM 7,8-DHF during 24 REOX following 4 h OGD recused female hippocampal neurons. However, this neuroprotection was lost when female hippocampal neurons were pretreated with T (Fig. 5B). This suggests a role for male androgens in regulating TrkB-dependent increases in ERα and subsequent neuroprotection in female hippocampal neurons. Interestingly, in male T pretreated hippocampal neurons we observed a small but significant increase in cell survival post-OGD/REOX with 7,8-DHF (0.22 ± 0.06 vs. 0.40 ± 0.04, *p* = 0.004).

## Conclusions

In this study, we investigated the role of ERα in mediating TrkB phosphorylation and neuroprotection in sexed hippocampal neurons following in vitro ischemia. Our results show that (1) Only in female ERα^+/+^ hippocampal neurons did 7,8-DHF increase TrkB phosphorylation and improve neuronal cell survival; (2) ERα was required for these sex-dependent TrkB mediated neuronal survival; (3) ERα mRNA expression was upregulated only in female hippocampal neurons following 4 h OGD and 3 h REOX; (4) TrkB antagonist, ANA12, blocked the in vitro ischemia induced increase in ERα mRNA expression in female neurons; 4) Pretreatment of female ERα^+/+^ hippocampal neurons with T blocked not only the in vitro ischemia induced increase in ERα mRNA expression, but also eliminated the sex differences in neuronal cell survival.

## Discussion

### In-vivo neuroprotective effects of 7,8-DHF

BDNF, acting through TrkB ^Y705^ activation, has been of interest as a therapeutic target because of its neurotropic actions in a number of neuronal populations [45]. However, BDNF has poor bioavailability in-vivo which has limited its use as a therapeutic agent [46]. On the other hand, small molecules with neurotrophin activity have recently been developed to overcome this issue. On such example is 7,8-DHF, which not only readily penetrates through the blood brain barrier when administered systemically, but robustly phosphorylates TrkB [15]. Consequently, 7,8-DHF administration has been used to improve outcomes in various models of neurological disorders including traumatic brain injury [47, 48], MCAO model of stroke [9], intracerebral hemorrhage [49], Parkinson’s disease [50], Alzheimer’s disease [51], retinal degeneration [52] and neonatal hypoxic ischemia [53]. However, the cellular mechanisms that result in 7,8-DHF dependent neuroprotection are poorly understood. Recently, we reported that in a mouse neonatal HI model the neuroprotective effects of 7,8-DHF are sexually differentiated and ERα dependent [7]. To further understand the sex and ERα dependent nature of 7,8-DHF neuroprotection we have extended that study to investigate 7,8-DHF neuroprotection in sexed hippocampal neuronal cultures following OGD.

### 7,8-DHF neuroprotection is linked to phosphorylation of TrkB

Here we report that administering 7,8-DHF to sexed hippocampal neurons results in a dose-dependent increase in p-TrkB ^Y705^ immunostaining with an ED_50_ of $\sim$ 2 µM that is more pronounced in female cells. Using purified TrkB proteins, Jang et al. reported that 7,8-DHF binds to the extracellular domain of TrkB. In receptor dimerization assays they found that 7,8-DHF initiates TrkB autophosphorylation. They concluded that while both BDNF and 7,8-DHF robustly bind and activates recombinant TrkB protein, the effect of BDNF is transient compared to 7,8-DHF where the effect can last hours [9, 15]. Tecuatl et al. reported that in adult rat hippocampal slices, application of 7,8-DHF resulted in phosphorylation of Trk-B in both CA1 and CA3 regions. In addition, when slices were pre-incubated with 7,8-DHF there was a marked downstream activation of the IP-3-K/Akt signaling cascade post-OGD/reperfusion [54]. In contrast, others have reported that in primary cortical non-sexed neurons and TrkB transfected HEK cells, 7,8-DHF failed to elicit phosphorylation of TrkB or its downstream effectors, ERK and AKT using sandwich ELISA [43]. Thus, it is possible that 7,8-DHF may initiate phosphorylation of TrkB indirectly in some systems.

Nevertheless, when 7,8-DHF is administered as a therapeutic agent in *in-vivo* models of neurological disease it results in TrkB phosphorylation in the hippocampus. In two-month-old male rats subjected to TBI, intraperitoneal injections of 7,8-DHF resulted in the restoration of hippocampal p-TrkB protein levels and p-TrkB immunohistological staining [55]. In addition, intrahippocampal injections of K252a, a TrkB antagonist, blocked the phosphorylation of TrkB by 7,8-DHF. Similar results were reported in juvenile male mice when 7,8-DHF was administered following a model of TBI and using ANA-12 as a TrkB antagonist [47]. We have reported that following HI injury in neonatal mice TrkB is phosphorylated in the hippocampus and that 7,8-DHF further enhances this increase in p-TrkB in female brains [7].

In addition to in-vivo models, 7,8-DHF has been shown to induce TrkB phosphorylation and neuroprotection in *in-vitro* systems. Jang et al. reported that 7,8-DHF application induces TrkB phosphorylation and decreases apoptosis following OGD/REOX in a dose dependent manner in non-sexed cultured hippocampal neurons [9]. When 7,8-DHF is administered to primary cultured non-sexed mouse motor neurons it results in activation of TrkB and increased cell survival [56]. In a similar manner, we found that OGD/REOX increases p-TrkB phosphorylation only in female hippocampal neurons and that administration of 3 µM 7,8-DHF post-OGD enhances this TrkB phosphorylation further and rescues cell survival in female neurons. Taken together, it is clear that in both *in-vitro* and *in-vivo* systems TrkB is phosphorylated in presence of 7,8-DHF resulting in profound neuroprotection. Interestingly, we also found that the phosphorylation of TrkB via 7,8-DHF is absent in hippocampal neurons cultured from ERα^−/−^ mice indicating that ERα is required for 7,8-DHF dependent TrkB signaling.

### ERα and TrkB-mediated sexually differentiated neuroprotection

Numerous rodent studies have shown that adult females have a lower incidence of naturally occurring stroke and are less sensitive to the damaging effects of focal or global ischemia injury [3]. The enhanced neuroprotection seen in adult females is generally proscribed to increased levels of circulating steroid hormones. However, this mechanism is not likely to be relevant in neonatal models of brain injury where there are minimal levels of circulating estrogen. Comparatively, few studies have investigated sexually dimorphic neuroprotection in models of neonatal brain injury. In a neonatal (P10) mouse HI model, the infarct size in male mice at 3 days following HI brain injury is larger than in females, and tissue loss at 30 days after HI is larger in male as compared to female mice. This result was attributed to increase microglial activation and inflammation seen in male neonatal brains 3 days following HI [57]. In our model of neonatal (P9) mouse, we did not observe a significant difference in male and female brain injury scores 3 days following HI. However, 7,8-DHF administration did result in a profound neuroprotective effect in female brains, both acutely and in long term behavioral studies [8].

Heyer et al. exposed sexed primary hippocampal neurons to either 15 h of OGD or normoxia [31]. Under normoxic conditions they reported that male hippocampal neurons exclude Trypan-blue at a rate slightly higher than females (83% male vs. 80% female). However, after 15 h of hypoxia cell death increases significantly more in female (182%) hippocampal neurons than in male neurons (129%) [31]. They did not investigate the effect of REOX on sex-dependent cell death. In sexed isolated primary neurons, there was no difference in male and female cells under control conditions or after cell death was induced by glutamate toxicity [58]. However, pretreatment with estradiol only protected female cells from glutamate toxicity, not male cells. In addition, the ERα agonist PPT promoted female neuroprotection while the ERα antagonist MPP blocked estradiol neuroprotection in female cells [58]. We did not observe a significance difference in cell death between male and female hippocampal neurons under either normoxic or OGD/REOX conditions. However, we did find that 7,8-DHF application results in a profound decrease in the OGD/REOX-induced cell death in female hippocampal neurons, but not in male neurons. Even more interesting, this effect was dependent on the presence of ERα. Taken together, this suggests a role ERα in the sexually dimorphic response to cellular stress in neurons.

Given that both 7,8-DHF dependent post-OGD TrkB phosphorylation and post-OGD survival in female hippocampal neurons require ERα, it implicates TrkB-ERα signaling as critical for this sexually differentiated response. Studying schizophrenia pathogenesis, Wong et al. reported that ERα and TrkB isoform converge to regulate ERα mediated gene transcription in the SHSY5Y neuronal cell lines [59]. Not only does BNDF have a variety of pro-survival neuronal functions, but the pleiotropic effects of BDNF are known to include sex specific regulation of its signaling [60]. More interesting, Solum and Handa have reported that ERα and BDNF are colocalized in the developing rat pup and BDNF mRNA expression was regulated by estradiol [61]. It has also been shown that ERα positive cells are particularly located in pyramidal neuronal layer of hippocampi [62].

Sexually differentiated increase of ERα mRNA expression in females following adult stroke or neonatal HI was previously described in rodent models [7, 23]. Heyer et al. reported that 15 h of hypoxia in primary hippocampal neurons results in an increase in ERα mRNA expression in female cells but not male cells. Estradiol treatment during hypoxia did not further enhance ERα mRNA expression in female cells [31]. In this study, we found that ERα mRNA expression increases following OGD/REOX in female hippocampal neurons with or without 7,8-DHF treatment. We also found that this sex specific OGD/REOX mediated ERα mRNA expression was blocked in the presence of the TrkB antagonist ANA12, suggesting that there is a crosstalk between ERα and TrkB signaling. Although male mice were not tested, a similar crosstalk between ERα and TrkB which is activated by 7,8-DHF has been reported to play a role in alleviating metabolic syndrome (MetS) in aged female mice [63]. Further investigation will be needed to elucidate how the interplay ERα and TrkB contributes to sex-specific neuronal survival following hypoxic insults.

### T and sexually differentiated Neuroprotection

There are numerous reports concerning the role of gonadal hormones in sex-dependent neuropathology using adult and juvenile rodent stroke models [3]. In adult rat middle cerebral artery occlusion models, removal of androgens by castration in males reduces stroke volumes with subsequent T replacement therapy resulting in increased brain damage [64, 65]. These results have been recapitulated in a mouse hippocampal cell line where T treatment significantly increased glutamate-mediated cell death [66]. It is well established that the perinatal “surge” of T in males is linked to the developmental masculinization of male neural morphology and behavior [30]. However, there are few studies on the effect of the surge in T in males on neonatal HI. In sexed hippocampal neuronal cultures, male neurons are more vulnerable to GABA-mediate injury than female neurons. Pretreatment with T caused increased cell death in both male and female neurons [67]. When female hippocampal neurons were treated with T for 30 min before 15 h of hypoxia, cell death was increased compared to hypoxia alone. The effect of T was reversed in the presence of estradiol [31]. T mediated TrkB regulation has also been reported. Administration of T has been shown to downregulate TrkB expression in the medial preoptic area (MPOA) in gonadectomized male hamsters [68]. These studies suggest that T changes the female brain neuroprotective phenotype to resemble the male brain.

We show here that in primary hippocampal neurons pre-treatment with T ablated the increase of ERα mRNA expression following OGD/REOX seen in female hippocampal neurons. Pretreatment with T also blocked the 7,8-DHF-dependent increase in female hippocampal neurons survival following OGD/REOX. Thus, in our study, T modifies the female neuroprotective phenotype making them more susceptible to OGD/REOX.

The mechanism involved in the T-dependent decrease in ERα expression is not clear and further investigation is needed. However, we did not find any sex difference in aromatase mRNA expression under normoxic conditions or following OGD/REOX in hippocampal neurons (data not shown). associated with an effect on genomic ERα [69]. Further investigation will be necessary to understand the role of T.

### Perspectives and significance

This study identifies a unique female-specific pathway of neuroprotection in hippocampal neurons that is inhibited in the presence of the male androgen T. We have shown in a particular cell type there is an intrinsic sex difference in the functioning and responsiveness to pharmacological agent which is an important finding given the need for effective therapies for HIE. In addition, further investigations that elucidate the role that androgens play in the male susceptibility to neonatal hypoxic encephalopathy could help to identify important therapeutic targets.

## Conclusions

**Fig. 6:**
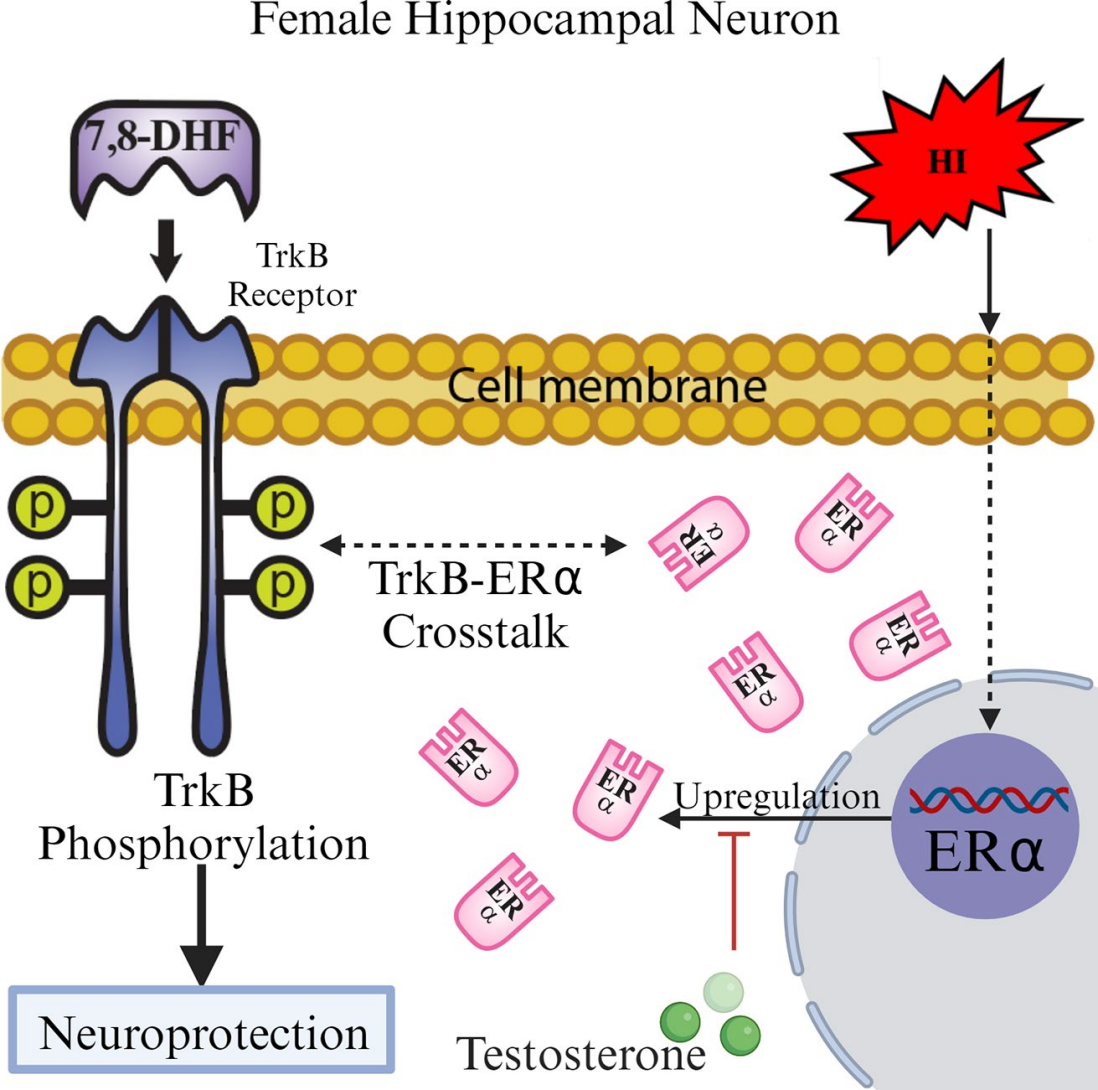
A model of female-specific neuroprotection in hippocampal neurons Hypoxia leads to increases in the ERα expression and responsiveness to 7,8-DHF in female hippocampal neurons. The resulting increase in TrkB phosphorylation promotes cell survival pathways in these cells. Pre-treatment with T blocks this pathway in a manner that has yet to be determined

In summary, we present evidence that support a new model for sex-dependent neuroprotection in hippocampal neurons following in vitro ischemia (Fig. 6**)**. In female neurons, in vitro ischemia results in TrkB activation an effect that is enhanced by the presence of 7,8-DHF and leads to an increase in ERα mRNA expression. Thus, cell survival is preferentially promoted in female hippocampal neurons. Interestingly, when female hippocampal neurons are pre-exposed to T this neuroprotective pathway is impaired (Fig. 6). Further investigation is needed to determine the mechanisms by which hypoxia increases ERα expression and how androgens may block this female specific neuroprotective pathway. Understanding these mechanisms could lead to better sex-specific targeted therapies and better clinical outcomes following neonatal HI.

## Data Availability

All data generated or analyzed during this study are included in this published article [and its supplementary information files].
